# “Nothing good happens after 2 am”: personal messages in a self-designed text-based alcohol intervention

**DOI:** 10.1186/1940-0640-8-S1-A57

**Published:** 2013-09-04

**Authors:** Karen A Renner, Ross McCormick, Natalie Walker

**Affiliations:** 1 Faculty of Medical and Health Sciences, University of Auckland, Auckland, New Zealand; 2 National Institute of Health Innovation, University of Auckland, Auckland, New Zealand

## 

Technology is enabling behaviour change interventions to incorporate text message reminders as a support to traditional intervention programmes, especially in the fields of smoking cessation, diabetes management, and weight loss. In this study, the intention was to trial a brief alcohol intervention which included web and mobile phone text elements enabling participants to create and schedule personal alcohol harm reduction messages. Two parallel-group randomized controlled feasibility studies, conducted between September 2011 and January 2013, attracted 136 visitors and 77 participants aged 18 to 67. Participants (54% European, 18% Māori) were predominantly female (63%) and half (48%) were college/tertiary students. They were randomly assigned to an intervention (participants could create their own text messages about their drinking and schedule the timing, number and frequency of the message delivery) or control (directed to standard alcohol information web sites) for three months. The primary outcome of each study was a change in unintended negative consequences from drinking at three months. Twenty four participants created 56 personal text messages. Messages that were neutral in tone employed a directive approach (e.g. “DO NOT PUNCH [name]”) providing pragmatic instructions on specific consumption reduction or safety actions. Fifteen abstract messages indirectly reminded the recipients of their safety intentions (e.g. “Don't get too rowdy ya silly girl…”) without specifying an action. Aspirational concepts (e.g. “Remember you’re the boss, act that way”) were contained in ten messages and represented the broadest range of motivations to encourage safe drinking. Sixty percent of the message content was very personal. Engaging participants in creating their own messages reveals a diversity of content approach and style which bears little resemblance to brief intervention messages commonly used in the alcohol brief intervention field. Empowering, personal messages delivered via text may resonate more deeply with the drinking population and improve alcohol brief intervention outcomes.

